# Neuroprotective nutrients in pregnancy and infant brain function

**DOI:** 10.1016/j.clnesp.2025.05.030

**Published:** 2025-05-22

**Authors:** D.N. Christifano, K. Liao, N.B. Mathis, S.E. Carlson, J. Colombo, L. Chollet-Hinton, K.M. Gustafson

**Affiliations:** a Department of Dietetics and Nutrition, University of Kansas Medical Center, Kansas City, KS 66103, USA; b Department of Neurology and Hoglund Brain Imaging Center, University of Kansas Medical Center, Kansas City, KS 66103, USA; c Department of Psychology and Schiefelbusch Institute for Life Span Studies, University of Kansas, Lawrence, KS 66044, USA; d Department of Biostatistics, University of Kansas Medical Center, Kansas City, KS 66103, USA

**Keywords:** Maternal diet, Infant neurodevelopment, Choline, Docosahexaenoic acid

## Abstract

Neuroprotective nutrients including omega 3 fatty acid docosahexaenoic acid (DHA), choline, and carotenoids play an important role in infant brain structure and function; however, the effects of maternal nutrition on the infant brain are understudied. We leveraged data from our large randomized clinical trial among pregnant women to determine whether maternal nutrients including DHA, choline, lutein/zeaxanthin (L/Z) and egg intake (a food source of these nutrients) were related to infant brain function. Data from n = 241 maternal and infant dyads were included in this secondary analysis. Food frequency questionnaires (Diet History Questionnaire II) at 32 weeks’ gestation were used to assess choline and carotenoid intake and a brief survey on egg consumption (# eggs consumed per week) was used to assess egg intake. Maternal red blood cell DHA status was measured at 32 weeks’ gestation. Infant brain function was measured using high density electroencephalogram (EEG) to auditory (1 month of age) and visual (6 months of age) event-related potentials (ERP). At 1 month of age, maternal DHA status was associated with greater delta-band spectral power to the novel tone during the ERP. Choline, choline*L/Z, L/Z*DHA, and choline*L/Z*DHA were each related to power to the frequent tone during the ERP. At 6 months, choline, DHA status, and choline*DHA were related to shorter latency to the novel visual stimulus. Choline*L/Z and choline*L/Z*DHA were related to greater amplitude to the novel stimulus. Overall, maternal neuroprotective nutrients were related to several markers of infant brain function.

## 1. Introduction

The fetal brain requires nutrients to grow and develop throughout gestation, and the availability of these nutrients depends on maternal intake. Docosahexaenoic acid (DHA), an omega 3 fatty acid, choline an important micronutrient and b-like vitamin, and carotenoids are all implicated in fetal and infant neurodevelopment. DHA is the most prevalent fatty acid in the frontal cortex, necessary for its function, and maternal supplementation has been associated with improved neurodevelopment among infants [1]. Choline is crucial for the formation of acetylcholine, a neurotransmitter that helps to establish and maintain neural networks throughout the lifespan [2] and has been linked to infant cognitive outcomes [3]. Carotenoids are well-studied for their role in brain health in aging populations; however, two specific carotenoids, lutein and zeaxanthin (L/Z) have recently been reported as the primary carotenoids found in the infant brain [4]. All of these nutrients play individual and synergistic roles in both structure and function of the brain.

While the importance of nutrition for brain development is evident, women are under-consuming neuroprotective nutrients during pregnancy. As many as 90 %–95 % of reproductive-aged women in the United States are not meeting recommendations for choline [5,6] or DHA intake [7]. While there are no global recommendations or Dietary Reference Intake (DRI) values set for carotenoids, reported intakes in pregnant populations are variable and likely insufficient [8]. Low intake of these nutrients is due in large part to the fact that standard prenatal vitamins do not commonly contain them, and women tend to under-consume food-rich sources such as seafood and eggs during pregnancy [9,10]. DHA is found most abundantly in fatty ocean fish (e.g. salmon) and in smaller amounts in foods such as eggs. Egg yolks also contain significant levels of choline and moderate levels of carotenoids [11]. In an analysis of the National Health and Nutrition Examination Survey (NHANES) data, people who consumed eggs had double the choline intake as those who did not consume eggs [6]. In pregnancy, women are more likely to meet the Adequate Intake (AI) of 450 mg/day for choline in pregnancy when consuming eggs or taking a supplement [6].

We reported previously that maternal nutrition during pregnancy, specifically a synergy of choline, L/Z, and DHA, was positively related to fetal neurodevelopment [12]. Here, we investigate whether those effects continue during infancy by evaluating infant brain function as Event Related Potentials (ERPs) via high density electroencephalogram (EEG) at 1 and 6 months of age in a cohort of 241 maternal/infants dyads.

## 2. Methods

**Fig. 1.**
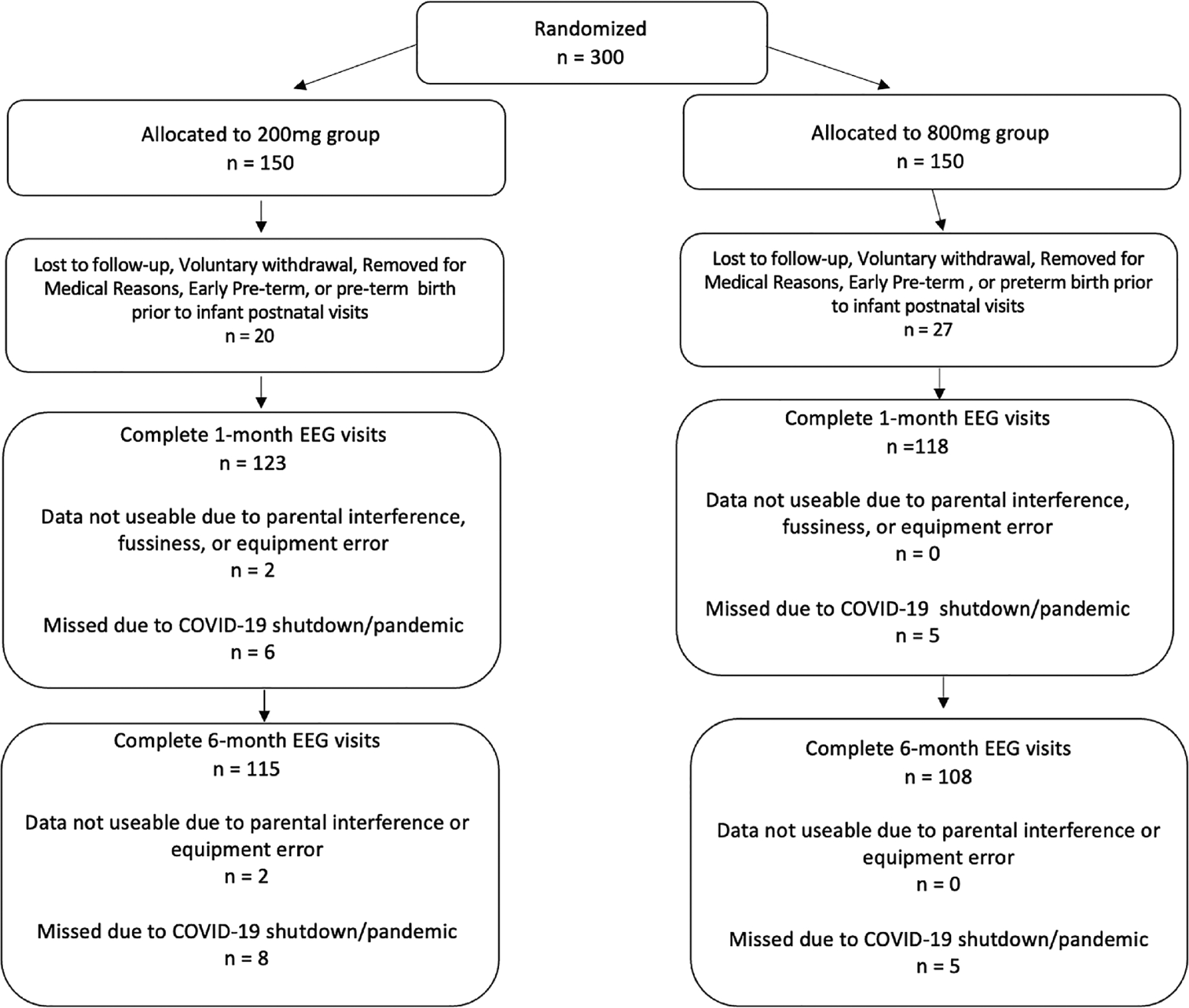
CONSORT diagram.

This study is a secondary analysis of the PANDA study [13], an NIH funded (HD086001) randomized controlled clinical trial examining the effects of 2 doses (200 mg and 800 mg) of docosahexaenoic acid (DHA) supplementation in pregnancy. Participants (n = 300) in the parent trial were randomly assigned (1:1) to take 200 mg (n = 150) or 800 mg (n = 150) of DHA daily beginning between 12 and 20 weeks of pregnancy and ending at delivery. Mean gestational age at enrollment in the PANDA sample was 18.0 ± 2.0 weeks. Of those enrolled in the parent trial, 47 did not complete postnatal follow-up due to being lost to follow-up (n = 14), voluntary withdrawal (n = 16), involuntary withdrawal (n = 5), early pre-term birth (n = 3), or pre-term birth (n = 9) (see Fig. 1). Specifically, this secondary analysis examines the effects of maternal choline, L/Z, and DHA (using DHA status after several months of DHA supplementation) or egg intake on ERPs at 1 and 6 months of age.

### 2.1. Measures

#### 2.1.1. Diet survey

*The National Cancer Institute Diet History Questionnaire II (DHQ-II)* is a food frequency and portion size questionnaire. The database associated with the DHQ versions are based on the National Health and Nutrition Examination Surveys (NHANES) data collection from 2001 to 2006. At 32 weeks, subjects completed version 3 (past month, with portion size) in order to determine diet during pregnancy. The survey was completed online by all subjects. Carotenoids including L/Z and choline intake were quantified from this survey as mg/day.

##### DHA-rich foods questionnaire:

A validated DHA-rich food questionnaire was administered at the enrollment study visit (gestational age at enrollment = 18.0 ± 2.0 weeks) [14]. Women were asked to identify from a list of DHA-containing foods which they consume (including number of egg yolks eaten per week) and the approximate frequency and portion size of each. Egg intake per day was quantified from this survey.

##### RBC:DHA Status:

Maternal blood was collected at 32 weeks using EDTA tubes, kept on ice, and processed with 24 hours to separate plasma, buffy coat, and RBCs. Samples were stored at −80°C. RBC phospholipid fatty acids, including DHA (reported as wt % of total fatty acids), were measured by gas chromatography using an Agilent 6890N system; further methodological details are available elswhere [13].

#### 2.1.2. Infant auditory and visual EEG and ERP

At 1 and 6 months postnatal age, infants underwent auditory and visual ERPs, respectively. EEGs were recorded using a high-density system (Magstim EGI, Oregon, USA). The purpose of the EEG protocols was to determine brain responses via ERP to auditory (1 mo) and visual (6mo) tasks. A sensor net containing 128 electrodes was soaked in saline, fitted to the infant head, and fastened with a chin strap. Infants were placed in a car seat or on their parent’s knee in the tests. The Net Amps 400 amplifier was used with a bandwidth from DC to 2000 Hz and input impedance larger than 1 GΩ. The electrode impedance was kept less than either 50 KΩ or 100 KΩ [15]. EEG data were digitized at 1000 Hz and referenced to Cz.

In the offline process, EEG data were band-pass filtered between 0.5 Hz and 30 Hz in Magstim EGI NetStation Tools software with a roll-off frequency of 0.3 Hz. All other EEG analyses were done in MATLAB (Natick, Massachusetts: The MathWorks Inc.) using EEGLAB [16] and ERPLAB toolboxes [17]. Only sixty-four electrodes were used in the data analysis to reduce computational workload. The Artifact Subspace Reconstruction (ASR) method [18] from EEGLAB was used with its default setting to reject or correct noisy data periods for auditory and visual EEG data, respectively. The Reject Data Epochs function from EEGLAB was also implemented, setting the threshold at ± 250 uV with an improbable data limit of five standard deviations. Subsequently, the adaptive mixture independent component analysis (AMICA) [19] was employed to detect and remove various ocular and movement artifacts or cardiovascular signals with ICLabel [20] and SASICA [21] plugins. Continuous EEG data were segmented into epochs regarding stimulus onset from − 100 ms to 650 ms for auditory ERP and from − 100 ms to 500 ms for visual ERP. Each epoch was baseline-corrected. Signals from bad electrodes were interpolated using surrounding electrode data. All electrodes were off-line referenced to the linked mastoid average. Auditory and visual stimuli were designed using the E-Prime software (version 3.0, Psychology Software Tools, Sharpsburg, PA).

#### 2.1.3. Infant auditory EEG (1 month)

In the auditory test, 1000 tones were played in a random order with 84 % frequent tones (fundamental frequency: 1000 Hz) and 16 % novel tones (frequency: 750Hz). No two novel tones were played consecutively. Each tone was 100 ms and the time between tone onsets was 768 ms with a random jitter of 40~50 ms. Tones were played at 80 dB from speakers on either side of the car seat. The auditory test lasted for approximately 13 min. Sixteen frontal electrodes were used in the spectral analysis, including Fp1, Fp2, F3, Fz, and F4. The evoked power spectrum function from ERPLAB was used to calculate delta band power (0–4 Hz). There were 243 EEG datasets in the auditory task with 2 datasets unable to be used due to equipment error (n = 1) or parental interference (n = 1).

#### 2.1.4. Infant visual EEG (6 months)

In the visual test, 230 color images were displayed in a random order, in which a frequent image (a cartoon ball) was shown in 70 % of the presentations and different novel images (cartoon toys and animals) were shown in the remaining 30 % [22,23]. Each novel image was only shown once, and no two novel images were presented successively. The duration for each image was 500 ms, preceded by a fixation from 600 to 1000 ms. The local peak amplitude and latency of the negative central (NC) ERP component [23] were measured as the most negative value in a 20 ms sliding window from 400 to 500 ms after the visual stimuli presentation. The visual test lasted about 5 min. There were 245 datasets in the visual task with 2 visits unable to be used due to equipment failure (n = 1) or parental refusal (n = 1).

#### 2.1.5. Statistical analysis

Simple descriptive statistics (means/standard deviations or frequencies/percentages) were tabulated for all variables. Scatter plots and histograms were used to investigate the adequacy of the model assumptions. SAS 9.4 M8 (TS1M8) was used for all analyses and statistical significance was set at α = 0.05. All tests were two-tailed, and *p* < 0.05 was considered statistically significant.

We used linear regression models to determine the relationship between ERP variables at 1 month (band-limited spectral power) and 6 months (latency and amplitude) to novel and frequent stimuli and both choline intake and egg intake. We also tested for the potential interaction of maternal dietary L/Z intake and maternal RBC DHA with choline and egg intake. For each model, we assessed the primary effects of each predictor—namely choline or egg consumption, dietary lutein/zeaxanthin (L/Z) intake, and red blood cell (RBC) DHA—as well as all possible two-way and three-way interactions among them. Interaction terms that were not statistically significant were progressively removed in a stepwise fashion to arrive at the final model. To facilitate comparison of the relative strength of both main effects and interactions on the outcome variables, all predictor variables were standardized using z-score transformation, and standardized regression coefficients were subsequently calculated. We also tested for confounding effects of other environmental factors known to be associated with the outcome measures; socioeconomic status (income), maternal education and smoking status. None of the models were significantly altered with the inclusion of these variables, so we did not report on these covariates.

## 3. Results

Descriptive statistics are listed in Table 1. For the EEG measures, 243 infants completed the 1 month visit and 225 infants completed the 6 month visit. Missed visits due to the institutional COVID-19 shutdown and/or participant COVID exposure or related worries totaled11 visits at 1 month and 13 visits at 6 months.

Results for 1-month ERPs are shown in Table 2. Neonatal EEG is dominated by delta frequency, hence the choice of this band-limited frequency [24]. In general terms, spectral power is a reflection of amplitude, e.g., the spatial summation of postsynaptic potentials derived from local synchronous activation of a large neuronal network. Therefore, greater power is thought to be an indication of a more coherent, synchronous neuronal response to the auditory stimuli [22]. Delta-band (0–4 Hz) spectral power to the novel stimulus was related to DHA status (*p* = 0.028); greater maternal DHA status at 32 weeks was related to greater Delta-band power. Delta-band power to the frequent stimulus was related to choline*L/Z (*p* = 0.003), LZ*DHA (*p* = 0.034), and choline*L/Z*DHA (*p* = 0.008). Egg intake alone was not related to our ERP variables of interest at 1 month.

Results for the 6-month ERPs are outlined in Table 3. Contrary to our hypothesis, there was no effect of maternal diet on the habituation response (peak ERP amplitude, latency) to the more frequent, repeated visual stimulus. However, choline intake was related to ERP latency to the novel pictures (*p* = 0.020), such that, faster ERP peak response time to novel visual stimuli was observed in infants born to mothers with higher choline intake. DHA status (*p* = 0.050) and choline*DHA status (*p* = 0.037) were also related to shorter ERP latency to the novel pictures. Interactions between choline*L/Z (*p* = 0.040) and choline*L/Z*DHA (*p* = 0.029) related to the amplitude of the novel pictures. Typically, in ERP experiments using frequent vs. novel stimuli, the response to novel stimuli has faster latency and greater amplitude than the response to the frequent stimulus. In this study, infants responded typically, but the response to the novel stimulus was enhanced by maternal choline, L/Z, and DHA.

## 4. Discussion

The infant ERP results reveal several significant relationships between infant performance during the ERP at both 1 and 6 months of age and maternal nutrients. At one month, infants born to mothers with higher DHA status showed higher delta-band spectral power in response to the novel stimulus. This finding is consistent with the interpretation that higher DHA status during pregnancy predicts more coherent, synchronous neural response in infants as young as 1 month of age. It is important to note that there were no differences between brain outcomes at 1 or 6 months of age in the DHA supplementation group analyses. These relationships held true even after examination of nutrient synergy between choline, L/Z, and DHA; however, no other single nutrient aside from DHA was predictive of these outcomes. In this study, 6 month old infants born to mothers with both higher intake of choline and DHA status had faster ERP peak response times to the novel stimulus at 6 months of age. Furthermore, interactions between choline*L/Z and choline*L/Z*DHA were related to amplitude to the frequent stimulus at 6 months, suggesting greater neuronal synchrony. These results suggest more rapid maturation (myelination) in infants with more prenatal exposure to choline, as ERP latency typically decreases with increasing age [24]. Because ERP latency decreases with maturation, these results suggest better CNS integrity reflecting possible increased myelination and synaptic pruning. Interestingly, we found similar nutrient synergy was related to *fetal* neurodevelopment in the same cohort [12], suggesting brain changes are sensitive to nutrients in utero and extend into infancy.

To examine the effects of maternal nutrition on infant brain function, we utilized high density EEG to examine ERPs. ERPs can be used to study cognitive processes (brain function) in infants [22] yielding information regarding latency, amplitude and patterns of brain activation. We used the NC ERP component in this study, which was reported to encompass both memory and attention mechanisms for learning and information processing [23]. Studies investigating the effect of maternal nutrient intake on infant brain function are limited but indicate a potential for long-term functional benefits of supplementation during critical periods of brain development. To our knowledge, only one study measured infant brain electrophysiology in a randomized trial where pregnant women were assigned to placebo or choline supplementation from the 2nd trimester to delivery. The investigators compared the infant auditory P50 ERP inhibition ratio between supplemented and placebo groups [25]. All infants demonstrated the typical suppression of the P50 response to paired auditory stimuli, i.e., the effect of habituation to paired stimuli, or inhibition of the response to the 2nd tone in a pair. The reduced amplitude P50 to the 2nd tone in a pair is a developmental feature that develops in fetal and early postnatal life.

Currently, there is no Dietary Reference Intake value set for DHA or carotenoids, and the AI for choline in pregnant women is based on requirements observed to prevent liver damage in adult men and was adjusted for weight-based factors to account for the pregnant woman and fetus [26,27]. The current AI for choline in pregnant women is 450 mg/day, but a recent meta-analysis showed adequate maternal choline intake during pregnancy and early postpartum, defined as a minimum of 550 mg/day and ranging up to 1g per day, was related to improvements in child neurocognition (e.g. memory, attention, and visuospatial learning) while deficiency (less than 550 mg/day) was related to a higher odds of neural tube defects [1,3]. Additionally, a few well-cited longitudinal studies of choline supplementation have shown amounts as high as 930 mg/day might be needed in pregnancy in order to see benefits in the child’s cognition up to 7 years of age [28,29]. There are numerous studies, including a Cochrane review, stating there is high quality evidence for DHA supplementation in pregnancy to prevent early preterm birth and a low-level evidence, meaning further research is needed, to support cognitive effects in offspring [30]. These studies have laid important groundwork for determining whether the Dietary Reference Intakes (DRI) in pregnancy deserve to be established for DHA, carotenoids, and reevaluated for choline.

In this secondary analysis of a large cohort of pregnant women, maternal intake of neuroprotective nutrients were related to infant brain function at both 1 and 6 months of age. While consumption of these nutrients during pregnancy is thought crucial for neonatal brain development, most standard prenatal vitamins contain no or very little quantities of such nutrients. Therefore, dietary intake from food (e.g. seafood and eggs) is necessary and guidelines regarding food and supplement intake during pregnancy are profoundly needed. Future research should focus on examining relationships between maternal choline, L/Z, and DHA intake and serum markers of these nutrients on fetal and infant neurodevelopmental outcomes through randomized controlled trials in large cohorts.

## 5. Limitations

The DHQ-II and DHA-FFQ are validated dietary assessment tools; however, there are limitations in collecting self-reported dietary intake data such as under or overreporting. The version of the DHQ-II reflected dietary intake over the past month and is not representative of the diet during the entirety of pregnancy. Additionally, of the 300 participants originally enrolled in the parent trial, only 265 were retained for postnatal follow up. This trial was conducted partially during the COVID-19 pandemic and as such, some postnatal visits were missed due to institutional shutdown, COVID exposure, or participant concern. While the number of missed visits was minimal given the extenuating circumstances, the present analyses are secondary in nature, and as such, are more vulnerable to type I and II errors.

## 6. Conclusion

Our results highlight the importance of maternal nutrition, specifically, neuroprotective nutrients like DHA, choline, and carotenoids (L/Z) on the offspring brain development and function. These data suggest that there is a synergistic relationship between DHA, choline, and L/Z leading to more efficient, synchronized neural processing at 1 month of age and faster neural processing at 6 months of age. Our findings reiterate the importance of maternal nutrition and the need to revise dietary recommendations during pregnancy to assure these crucial nutrients are available to the developing fetus. Future research should further examine this relationship as well as optimal consumption of these nutrients during pregnancy to promote pre-and postnatal brain development.

## Figures and Tables

**Table 1:** Descriptive statistics.

Maternal	
Total (n)	300
Age at enrollment (yrs)	30.3 ± 4.9
Maternal race and ethnicity, n (%)	
American Indian or Alaskan native	2 (0.7)
Asian	11 (3.7)
Black or African American	39 (13.0)
Hispanic	37 (12.3)
Native Hawaiian or Pacific Islander	1 (0.3)
White	203 (67.7)
Biracial: Asian, White	2 (0.7)
Biracial: Black, native American	2 (0.7)
Biracial: Black, White	3 (1.0)
Multiracial: Black, native American, White	2 (0.7)
Maternal education, n (%)	
Less than high school graduate	6 (2.0)
HS Graduate or GED	29 (9.7)
Some college or tech school	73 (24.3)
Bachelor’s degree obtained	105 (35.0)
Master’s degree obtained	72 (24.0)
Doctorate	15 (5.0)
Family income, n (%)	
Less than $15,000	22 (7.3)
$15,000 – $24,999	16 (5.3)
$25,000 – $49,999	39 (13.0)
$50,000 – $99,999	102 (34.0)
$100,000 – $149,999	81 (27.0)
At least $150,000	38 (12.7)
Unknown	2 (0.7)
Ever smoker, yes n (%)	75 (25.0)
6 Months prior, yes n (%)	36 (12.0)
Current smoker, yes n (%)	12 (4.0)
Egg intake at baseline (eggs/wk)	4.7 ± 4.4
L/Z intake at 32wks (mcg/day)	2716.8 ± 3352.9
Choline intake at 32wks (mg/day)	275.1 ± 137.8
RBC DHA at 32wks (%^a^)	10.1 ± 2.6

Data are presented as mean ± standard deviation or n(%).

a % fatty acids of total fatty acid weight.

**Table 2:** Infant event-related potential outcomes (power) to frequent (A) and novel (B) tones at 1 month and relationships with neuroprotective nutrients and eggs.

Variable	Beta (SE)	95 % Confidence Interval	p-value
**Power (frequent; delta 0–4Hz)**			
Choline intake (mg/day)	−0.004 (1.362)	(−2.673, 2.665)	0.387
L/Z intake (mg/day)	−0.10 (0.394)	(−0.872, 0.672)	0.011
DHA status (% TFA)	−0.037 (0.125)	(−0.282, 0.208)	0.765
Choline*L/Z	0.003 (0.001)	(0.001, 0.005)	0.003
Choline*DHA	0.0001 (0.0004)	(−0.0007, 0.0009)	0.753
L/Z*DHA	0.072 (0.034)	(0.006, 0.138)	0.034
Choline*L/Z*DHA	−0.0002 (0.0001)	(−0.0004, 0.0000)	0.008
**Power (novel; delta 0–4Hz)**			
Choline intake (mg/day)	0.004 (0.003)	(−0.002, 0.010)	0.137
L/Z intake (mg/day)	0.009 (0.026)	(−0.042, 0.060)	0.724
DHA status (% TFA)	0.155 (0.071)	(0.016, 0.294)	0.028
Choline*DHA	−0.001 (0.0004)	(−0.002, 0.000)	0.055
**Egg model**			
Egg intake (eggs/week) (frequent)	−0.011 (0.113)	(−0.233, 0.211)	0.926
Egg intake (eggs/week) (novel)	−0.038 (0.094)	(−0.223, 0.147)	0.687

Linear regression models between ERP power and 1) choline, L/Z, DHA, and 2) eggs.

**Table 3:** Infant event-related potential outcomes at 6 months (amplitude to frequent (A) & novel (B) and latency to frequent (C) and novel (D) tones) and relationships with neuroprotective nutrients and eggs.

Variable	Beta (SE)	95 % Confidence Interval	p-value
**Amplitude and latency analysis**			
**A. Amplitude (frequent)**			
Choline intake	0.005 (0.003)	(−0.001, 0.011)	0.125
L/Z intake	0.0004 (0.117)	(−0.229, 0.230)	0.997
DHA status	−0.085 (0.141)	(−0.362, 0.192)	0.548
**B. Amplitude (novel)**			
Choline intake	0.027 (0.026)	(−0.024, 0.078)	0.294
L/Z intake	3.229 (2.145)	(−0.963, 7.421)	0.134
DHA status	1.028 (0.746)	(−0.434, 2.490)	0.170
Choline*DHA	−0.002 (0.003)	(−0.008, 0.004)	0.424
Choline*L/Z	−0.008 (0.004)	(−0.016, 0.000)	0.040
L/Z*DHA	−0.328 (0.196)	(−0.712, 0.056)	0.096
Choline*L*/ZDHA	0.001 (0.0003)	(0.0004, 0.0016)	0.029
**C. Latency (frequent)**			
Choline intake	0.084 (0.050)	(−0.014, 0.182)	0.093
L/Z intake	−3.554 (2.053)	(−7.578, 0.470)	0.085
DHA status	1.671 (1.277)	(−0.832, 4.174)	0.192
Choline*DHA	−0.010 (0.005)	(−0.020, 0.000)	0.051
L/Z*DHA	0.319 (0.182)	(−0.037, 0.675)	0.081
**D. Latency (novel)**			
Choline intake	−0.096 (0.041)	(−0.176, −0.016)	0.020
L/Z intake	0.133 (0.488)	(−0.823, 1.089)	0.786
DHA status	−2.487 (1.260)	(−4.957, −0.017)	0.050
Choline*DHA	0.009 (0.004)	(0.001, 0.017)	0.037
**Egg model**			
Amplitude (frequent)	−0.010 (0.076)	(−0.159, 0.139)	0.900
Amplitude (novel)	0.937 (0.513)	(−0.068, 1.942)	0.070
Latency (frequent)	−0.036 (0.302)	(−0.628, 0.556)	0.906
Latency (novel)	0.467 (0.410)	(−0.337, 1.271)	0.256

Linear regression models between ERP amplitude and latency ERP at 6 months and 1) choline, L/Z, DHA, and 2) eggs.
